# Dominique-Jean Larrey (1766-1842): The Founder of the Modern Triage System

**DOI:** 10.7759/cureus.62375

**Published:** 2024-06-14

**Authors:** Matthew D Turner, Muhammad Hamza Shah

**Affiliations:** 1 Emergency Medicine, Penn State Health Milton S. Hershey Medical Center, Hershey, USA; 2 Deanery of Biomedical Sciences, The University of Edinburgh, Edinburgh, GBR; 3 School of Medicine, Dentistry and Biomedical Sciences, Queen's University Belfast, Belfast, GBR

**Keywords:** military trauma, ambulance services, emergency medical service, trauma surgery, historical vignette

## Abstract

Dominique-Jean Larrey was a prominent French surgeon who rose to fame during the age of the Napoleonic Wars. During his service in the French military, he developed dozens of medical innovations. Most important of all were his improvements to the evacuation of the wounded from the battlefield, triage of the wounded, and rapid surgical intervention. His innovations revolutionized military medicine and are still the basis for modern practice today.

## Introduction and background

Introduction

**Figure 1:**
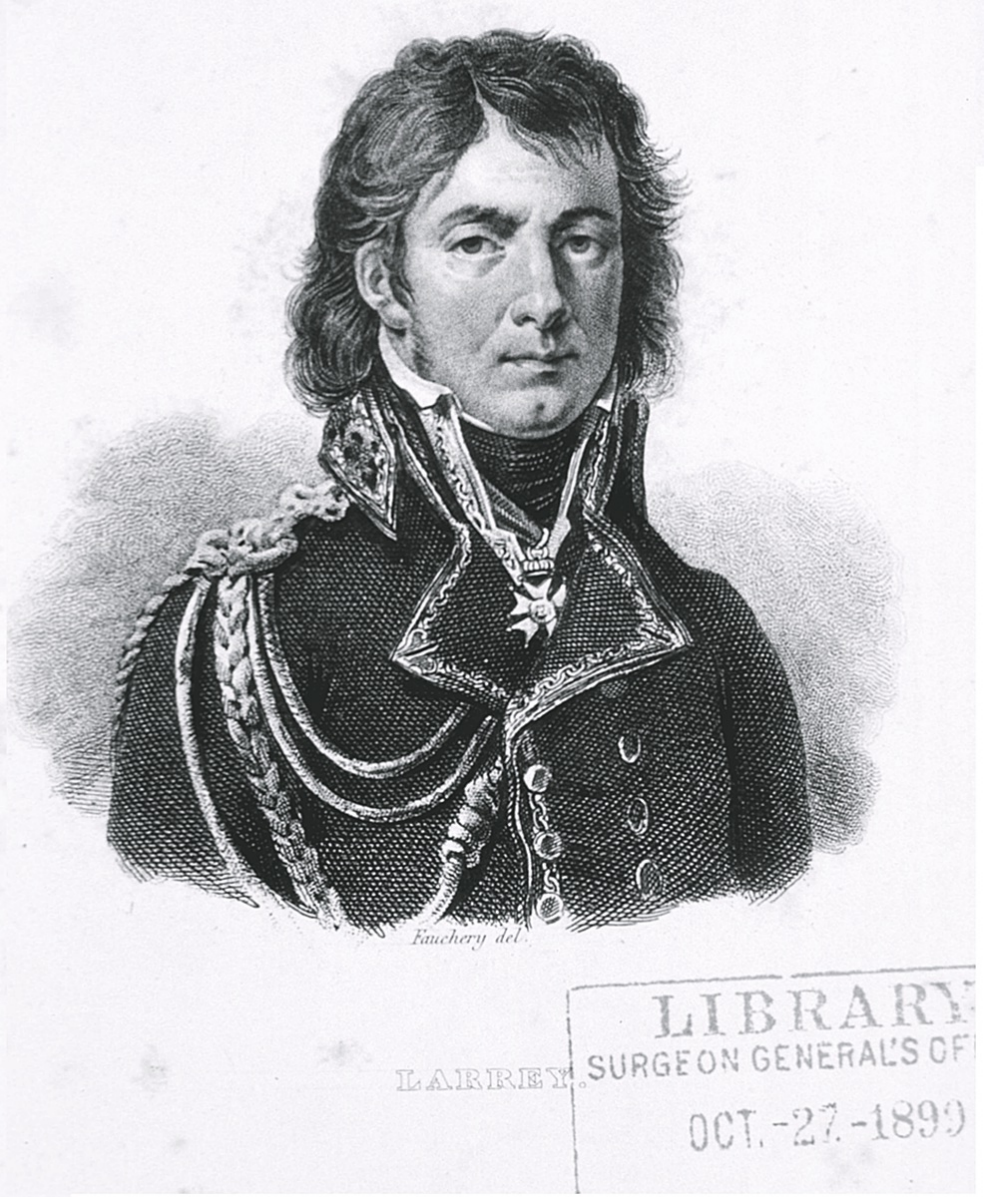
Portrait of Dominique-Jean Larrey Image obtained from the National Library of Medicine, published under Creative Commons 1.0 - Public Domain [1].

The contributions of Dominique-Jean Larrey (Figure 1) to modern medicine are too numerous to account in a single article. As a battlefield surgeon during the Napoleonic Wars, he is credited with an estimated 24 significant surgical advances [2]. He documented dozens of diseases ranging from tetanus on European battlefields to parasites in Egypt [3] and is credited as both the “father of modern military medicine” and “the father of emergency medical services” [4]. This article will focus on arguably his most important contribution to medicine: the modern ambulance and triage system [5]. Larrey’s innovations in these fields put the French army’s medical care decades ahead of its peers [2] and earned him the personal admiration of Emperor Napoleon Bonaparte, who described Larrey in his will as “the most virtuous man I have ever known” [6].

## Review

Larrey's life and career

Born in a small village in southern France in 1766, Larrey was orphaned at a young age. Throughout his early childhood, he was tutored by a local priest [7]. At the age of 13, he walked 70 miles alone to Toulouse to study medicine under his uncle's tutelage, where he began an apprenticeship as a “dresser” [7]. There, he took a particular interest in the dissection laboratory, and soon won renown among his peers, winning first prize in a medical competition at the age of 19 [5]. At the age of 21, he walked to Paris, where he underwent a rigorous examination and was appointed Auxiliary Surgeon in the French Navy. His first assignment was in 1788 to the frigate *Vigilante*. Larrey’s stint in the Navy did not last long. After the ship took him to Newfoundland, he developed severe sea-sickness, and resigned after only six months [7].

Upon returning to France, Larrey started work at the famous *Hôtel Dieu* and *Hôtel Royal des Invalides facilities* [7]. Meanwhile, the world was changing. Revolutionary fervor swept through France, and as the tumultuous wars of the period began, Europe witnessed a period of mass mobilization and war causalities on a scale that the continent had never before experienced [6]. Larrey’s first taste of war came in 1792, when he joined the Army of the Rhine as a *Chirurgien aide-major* [7]. Larrey’s administrative services served him well in this position, in which he acted as medical chief for a division of the army [8]. He observed that wounded soldiers on the battlefield would often not receive care for up to 36 hours, and proposed a model of ambulance volante, “flying ambulances” [8] inspired by the fast-moving horse artillery of the French Army [9]. Larrey’s ambulance system would employ two-wheeled and four-wheeled wagons pulled by teams of horses for rapid transport, as well as food, bandages, water, and the ability to employ “on the spot surgery” [5]. Eventually, the system was approved by Napoleon Bonaparte himself, who soon developed a close personal friendship with the physician [5]. Larrey’s abilities allowed him to swiftly climb through the ranks, and in 1805, Napoleon appointed him Surgeon in Chief of the French Army, as well as granting him the title of Baron in 1810 [6].

Over the course of the Napoleonic Wars, Larrey accompanied Napoleon in 25 campaigns that involved 60 battles and some 400 engagements [10] across Egypt, Palestine, Corsica, Spain, Germany, Poland, Russia, and multiple other theaters [6]. He developed innovations in hemorrhage control, debridement of wounds, rapid amputation, packing of sucking chest wounds, hemothorax drainage, aspiration of hemopericardium [2], a new design of surgical needle [8], and even a primitive form of cardiopulmonary resuscitation (CPR), performed by using a bellows to blow air into the lungs through the nares and then compressing the chest [7]. In the Russian campaigns, he observed that cold could be used as a potent analgesic and became a vocal proponent for this method [6].

Just as impressive as Larrey’s myriad medical innovations was his famous humanism. He earned the reputation as “father to the wounded” due to his empathy, care, and boundless energy for those under his charge. Napoleon himself noted, “If the army were to raise a column to the memory of anyone, it should be to that of Larrey… all the wounded are of his family” [11]. Larrey was so beloved by the army that at one point during the disastrous Russian campaign, a contingent of beaten and retreating French soldiers willingly risked their lives against enemy attacks to evacuate the surgeon, calling out “Save him who saved us!” [12]. He was fearless, often operating on active battlefields without taking cover or having any regard for “shot or shell” [11]. In one famous incident, as he applied a dressing to a soldier’s wound, a shell exploded almost directly above him; Larrey continued to focus on his patient and did not acknowledge how close he had come to death [12]. Larrey strongly advocated for a modern system of battlefield triage, where the wounded were treated by their severity of injuries without regard to rank [2]. He also treated enemy soldiers, earning him the recognition of enemy armies. Upon recognizing Larrey at the Battle of Waterloo, the Duke of Wellington ordered his men not to fire on the enemy physician treating the fallen wounded [3].

Larrey’s reputation ultimately saved his life. At the disastrous Battle of Waterloo, he was captured by Prussian soldiers. Only minutes away from execution, he was recognized by an enemy surgeon and brought to the Prussian commander. Larrey had saved the commander’s son’s life several years ago; in gratitude, the commander canceled the execution [11]. Larrey was soon released. In the wake of the war, he continued his medical service for another 27 years, further organizing medical services in both France and Belgium. Subsequent French governments recognized his achievements, confirming the status of Baron that Napoleon had given him [6]. He finally died in 1842 at the age of 76, “active to the end of his days” [6].

Triage and ambulances

Conventional military medical practice at the time of the Napoleonic Wars held that wounded on the battlefield should be left where they fell, and then gathered up at a single location following the conclusion of the battle [11]. At best, wounded soldiers had to rely on either self-evacuation or evacuation that relied on untrained and disorganized civilians [13]. Larrey observed that this often delayed care from 24 to 36 hours [11]. In many cases, the wounded would have to wait days to be treated, putting them at severe risk from complications from tetanus, sepsis, and other battlefield ailments [5].

Early on in his career, Larrey recognized the flaws in this system. Against typical medical practice, he was an advocate of surgery as swiftly as possible, and at the bare minimum demanded surgery within 24 hours of wounding [6]. “It is necessary to take advantage of the favorable moment to do the amputation,” he wrote, “Without waiting, against the advice of the majority of authors to wait until the dead tissue is well established” [5]. The unprecedented scale of the conflicts that he participated in necessitated swiftness and the ability to treat massive numbers of patients. Larrey was able to amputate a leg within 1 minute, and an arm in 17 seconds [6]. At the Battle of Borodino in 1812, he performed over 200 amputations in a single 24-hour period [5]. His development of the “inverted cone” approach to amputation, which allowed skin flaps to be sewn together over the site, as well as washing the wounds, significantly improved patient outcomes [6]. Larrey’s use of swift amputation, when compared to delayed amputation of a week or more, reduced mortality from over 90% to less than 25% [8].

**Figure 2:**
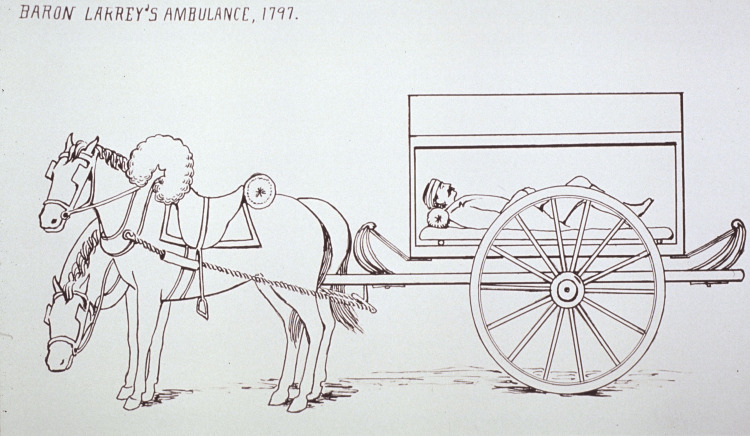
Larrey's two-wheeled "flying ambulance" Obtained from the National Library of Medicine. This image is published under Creative Commons 1.0 - Public Domain [14].

While Larrey’s personal skills as a trauma surgeon were impressive, his most significant accomplishment was his development of “flying ambulances”. While the British Army used primitive carts to transport their wounded, Larrey designed specialized transports - two-wheels (Figure 2) for flat terrain and four-wheels for rough terrain - that included medical supplies for swift surgery, food, dressings, and water to wash wounds [2].

These wagons were specially designed, and employed sliding shutters, spring suspension, padded litters, and doors at the front and rear to better facilitate patient transport [8]. Ambulance units were highly organized in units of three divisions of 113 men each, with a chief surgeon, 15 other surgeons, a trumpeter to carry surgical instruments, and a drummer boy in charge of dressings. Men in the ambulance units wore standard uniforms, and carried a saber for personal defense [2]. These ambulances swiftly took the wounded, both friendly and enemy, from active battlefields, providing emergency services on the way, and gathered casualties at a* chirurgicie de bataille* [8] located 3 miles behind the lines [13], comparable to modern military field hospitals [2]. Ambulance units were effective and highly adaptable - in the Egyptian campaigns, Larrey adjusted the system to the sandy desert terrain. Instead of using specialized wagons, ambulances employed camels, attaching a basket to each side of the camel that allowed the wounded to lay down. Each division was assigned 24 camels for medical evacuation [3]. In the grueling 1813 Russian campaign, these ambulance units were able to successfully adept local wheelbarrows for effective transport of the wounded [8]. At its peak, Larrey’s ambulances were able to reduce the delay in medical care for the wounded from the previous 24-36 hours down to approximately 1 hour [11].

Despite the impressive modernity of this system, other countries took decades to follow France’s example, still leaving the wounded on the battlefield. The British Army did not employ ambulances for another 60 years, preferring to abandon the sick or have them transported in local carts [15]. The United States did not begin evacuating wounded from active battlefields until after the Battle of Antietam in 1862 [2].

Once casualties were gathered at a field hospital, Larrey developed a remarkably modern triage system. Triage, which originates from the French *trier*, meaning “to sort”, was developed by Larrey in approximately 1792 [16]. He wrote, “We would always start with the most dangerously wounded without regard to rank or distinction,” and declared that “…those less severely wounded must wait until the gravely hurt have been operated and addressed,” a highly unusual innovation for the time [2]. The battlefield hospitals that the wounded were treated at were also stockpiled with large amounts of food, surgical supplies, and were sanitary, further innovations that Larrey developed [2]. Wounded soldiers in these hospitals received the most up-to-date care available at the time; surgeons employed Larrey’s innovative techniques of rapid amputation and thorough cleaning to significantly reduce gangrene and sepsis, dramatically improving patient survival [13].

## Conclusions

Among his many other accomplishments, Dominique-Jean Larrey was an exceptionally talented administrator and innovator. His improvements to battlefield evacuation and triage revolutionized military medicine and put the French army decades ahead of its contemporary peers. In addition to this, he invented dozens of new medical techniques that ultimately saved countless lives. Even during his lifetime, he was widely recognized for his life-saving achievements, and his innovations are still the basis for much of emergency medical services to this day.
